# Recurrence of nephrotic syndrome/focal segmental glomerulosclerosis following renal transplantation in children

**DOI:** 10.1007/s00467-006-0361-6

**Published:** 2007-04-01

**Authors:** Richard N. Fine

**Affiliations:** School of Medicine, Stony Brook University, Stony Brook, NY 11794-8111 USA

**Keywords:** Recurrent focal segmental glomerulosclerosis (FSGS), Plasmapheresis, Nephrectomy, Immunosuppressive medications, Long-term outcome

## Abstract

- The incidence of recurrence of nephrotic syndrome/focal segmental glomerulosclerosis (NS/FSGS) is variable (~30%)
- The incidence of recurrence is less in African-Americans than in whites and Hispanics
- Graft survival rates are decreased in recipients with FSGS, especially if remission of the NS is not achieved in those with recurrence
- Although controversial, the use of living donor (LD) transplants are not contraindicated; however, obligatory heterozygote parental grafts with a podocin mutation should be used with caution
- Optimal treatment to induce a remission post-transplant has not been delineated
- Pre-transplant and/or prophylactic post-transplant pre-operative plasmapheresis (PP) for high-risk patients—especially those with recurrence in a previous graft—may be promising
- An international multicenter controlled study is required to delineate the optimal approach to prevent and/or treat the recurrence of NS/FSGS

## Introduction

Focal segmental glomerulosclerosis (FSGS) is a histologic (pathologic) diagnosis that encompasses numerous specific clinical entities with potential varied etiologies. Steroid-resistant nephrotic syndrome (SRNS) is a specific clinical phenomenon that may vary histologically from minimal change nephrotic syndrome (MCNS) to FSGS, but it also may be associated with other specific histological diagnoses.

The most frequently acquired disease resulting in end-stage renal disease (ESRD) in children is FSGS, accounting for 8.3% of the chronic renal insufficiency (CRI) patients, 14.3% of the dialysis patients, and 11.4% of the transplant recipients included in the North American Pediatric Renal Transplant Cooperative Study (NAPRTCS) registry [1].

In 1972, Hoyer et al. [2] from Minnesota described the recurrence of NS in 3/4 pediatric renal allograft recipients whose original disease was FSGS. At post-mortem examination, the lesion of FSGS was present in the grafts of two recipients [2]. An editorial accompanying the publication of this article in *Lancet* stated that “renal transplantation may be contraindicated (in patients with FSGS) leaving dialysis as probably the only means of prolonging life” [3].

The purpose of this teaching review article will be to address the factors that influence the incidence of recurrence of NS/FSGS in renal allografts, establish the known risk factors for recurrence, and delineate the current preventive and therapeutic approaches to the recurrence of NS/FSGS in pediatric renal allograft recipients.

## What is the incidence of recurrence of post-transplant NS/FSGS?

The general consensus is that 30–40% of recipients whose original disease was NS/FSGS will clinically manifest recurrence [4]. In most instances, recurrence is clinically apparent in the immediate post-transplant period, frequently with substantive proteinuria in the initial 24 hours following transplantation. However, the onset of clinical recurrence has been reported to occur at more than one year following transplantation. The delay in initial clinical manifestations may be coincident to the occurrence of acute tubular necrosis (ATN), which occurs more frequently in live donor (LD) and deceased donor (DD) recipients with FSGS [5]. It has been proposed that the “immediate fulminate recurrence of NS/FSGS post-transplant likely predisposes such grafts to ATN and explains the increased incidence of ATN in this patient population [5].

### What is the incidence of subsequent recurrence in a patient who manifested clinical recurrence in a previous graft?

Re-transplantation of a recipient with recurrence of NS/FSGS in a previous graft has been associated with a ~80% recurrence rate [4]. Recently, a 100% recurrence rate has been reported in ten recipients of a second (6), third (3), and fourth (1) transplant [6]. Therefore, proceeding with a subsequent transplant in a patient who manifested previous recurrence has been discouraged. Recently, pre-operative plasmapheresis (PP) was successful in preventing clinical recurrence in 3/6 patients whose initial graft was lost in association with clinical recurrence [7].

### What is the incidence of recurrence in recipients with mutations of genes that encode for podocin?

The association of mutations impacting on the structure of the foot processes in the region of the slit diaphragm of the glomerular basement membrane in patients with sporadic and familial NS has potentially delineated the pathophysiology of the NS in such patients [8]. Initial publications indicated that the incidence of recurrence of NS/FSGS in patients with podocin mutations was similar to that reported for all patients with NS/FSGS [9]. However, a recent analysis of 73 patients with either homozygous/compound heterozygous (65) or heterozygous (8) mutations revealed a 7.7% incidence in the former and a 62.5% incidence in the latter recipients [10]. If these data are confirmed, it would seem advantageous to test all potential recipients with ESRD secondary to NS/FSGS for such mutations in order to delineate an optimal transplant strategy.

### Do patients with the glomerular tip lesion who receive a renal allograft manifest recurrence of the NS?

Howie et al. [11] reported that 12 of 14 recipients whose native kidney manifested the glomerular tip lesion had clinical recurrence of the NS post-transplant. In all 12 recipients, the glomerular tip lesion was the only histologic abnormality on graft biopsy. These data support the concept that the glomerular tip lesion may be the earliest histologic abnormality in the NS/FSGS spectrum of disease. Precise incidence and outcome data await subsequent reports.

### Does race impact on the recurrence of NS/FSGS?

Although the disease process appears more virulent in African-American (AA) children with NS/FSGS [12], it is interesting that the incidence of recurrence following transplantation in this ethnic population is decreased. At least three studies [5, 13, 14] have shown that the incidence of recurrence in the AA recipient was <50% of that in the Caucasian recipient. Hispanic recipients had an incidence similar to that of Caucasians. The precise reason for this disparity in incidence has not been delineated.

### Has the incidence of recurrence changed with the introduction of new immunosuppressive regimens?

Since the initial publication in 1972 of Hoyer et al. [2], there has been a significant change in the available immunosuppressive agents and regimens to both prevent and treat rejections. Despite these regimens, which have substantially reduced the incidence of acute rejection episodes and markedly improved short-term allograft survival rates, the incidence of recurrent NS/FSGS has remained unaffected [14]. Bartosh et al. [14] reviewed the NAPRTCS database for the period 1998–2003 and found the incidence of recurrence was 31%, which was similar to the incidence in the initial NAPRTCS report for the period 1987–1990 [4].

## What are the risk factors for the recurrence of NS/FSGS?

Three factors identified in the 1970s to be associated as risk factors for recurrence were: (1) the rapid progression to ESRD in a period of <3 years; (2) the presence of mesangial hypercellularity on biopsy; and (3) the age at onset of the clinical NS of >6 years. More recently, the presence of a circulating permeability factor [15], the use of induction therapy in the immunosuppressive regimen [16], and native kidney nephrectomy either prior to or at the time of transplantation [17] have been reported to be associated with an increased risk of recurrence.

### Does the presence of an identified circulating factor predispose to the recurrence of NS/FSGS?

Savin et al. [15] showed that the presence of a permeability factor (Palb) with an activity of ≥0.50 was associated with a recurrence rate of 86% (6/7), whereas an activity of <0.50 was associated with a recurrence rate of 17% (4/23). Plasmapheresis was associated with both a reduction in the Palb activity and in urinary protein excretion.

### Is induction therapy a risk factor for the recurrence of NS/FSGS?

Raafat et al. [16] noted a recurrence rate of NS/FSGS of 43% between 1968 and 1997. However, it was only 11% (1/9) in those who did not receive induction, whereas it was 53% (15/28) in those receiving some form of antilymphocyte serum (ALS). Conversely, Gagnodoux [17] reported an incidence of recurrence of 31% prior to 1985 when no induction therapy with antilymphocyte globulin/antithymocyte globulin (ALG/ATG) was used compared to 30% following the introduction of induction therapy. Both Sheth et al. [18] and Hubsch et al. [19] noted a significant increase in the incidence of recurrence (17.6% vs 37.5% and 38% vs 83%, respectively) with the use of anti-IL_2_ receptor antibody as induction therapy. In the most recent analysis of NAPRTCS for the period 1998–2003, Bartosh et al. [14] noted an overall incidence of recurrence of 31% (51/165), which was similar to no induction (30%) or the use of polyclonal antibody (27%) or monoclonal antibody (33%). Therefore, advocating the restriction of induction therapy to reduce the incidence of recurrence awaits the performance of a multicenter prospective randomized controlled study.

### Is native nephrectomy a risk factor for the recurrence of NS/FSGS?

Analysis of the recent NAPRTCS database [14] indicated a significant (*p* < 0.001) difference in the incidence of recurrence in recipients who had undergone prior native kidney nephrectomy (50%) compared to those whose native kidneys were not removed prior to or at the time of transplantation (22%). A possible explanation for this phenomenon was that the native kidneys provided a “sponge” for any circulating putative antibody and that native nephrectomy facilitated a sufficient level of circulation of such antibodies which were deposited in the graft precipitating recurrence. Other reports [20] have come to the same conclusion.

## Does the recurrence of NS/FSGS impact on the allograft survival rate?

Baum et al. [21] in 2002 and Bartosh et al. [14] in 2004 reviewed the NAPRTCS database, which indicated inferior long-term graft survival rates in both recipients of LD and DD grafts whose original disease was FSGS. Analysis of the UNOS database demonstrated a similar disadvantageous graft survival rate for recipients whose original disease was FSGS compared to recipients with other primary renal diseases [22].

A consensus is that 50% of the grafts with the recurrence of NS/FSGS are lost, presumably as a result of the recurrent pathologic process. However, data from Schachter and Harmon [23] noted that long-term survival rates significantly (*p* = 0.002) improved in those recipients with recurrence who achieved remission with some therapeutic intervention compared to those who failed to go into remission and had persistent NS.

## Does age at the time of transplantation impact on graft survival in recipients with FSGS?

Adolescent recipients (13–17 years of age) with FSGS have a significantly poorer graft survival rate with both LR and DD grafts compared to younger recipients (0–12 years of age) [21]. In actuality, the 0–12 year-old recipient DD graft survival rate was similar in the FSGS and no FSGS groups. The specific reason for this phenomenon is not known. However, Scientific Registry of Transplant Recipients (SRTR) data have consistently demonstrated that adolescent recipients have substantially poorer long-term graft survival rates, which approach that of recipients >65 years of age at transplantation [24].

## Should an LD graft be utilized in children with ESRD secondary to FSGS?

At least five reports from 1989 to 1999 [4, 25–28] concluded that recipients with FSGS had a greater risk of allograft failure from an LD graft than from a DD graft. A precise reason for this phenomenon was not delineated.

In 2001, Baum et al. [5] reviewed the NAPRTCS database and showed that the 5-year LD graft survival rate was 69% for recipients with FSGS compared to 82% with no FSGS (*p* < 0.001), whereas it was 60% and 67%, respectively, in the DD groups. The authors concluded that the long-term advantage of LD grafts was lost in recipients with FSGS.

Conversely, Huang et al. [22] proffered that, although the LD advantage was not observed in AA pediatric recipients, the authors recommend that LD transplantation be considered for all racial groups with FSGS.

With this potential excellent outcome with newer preventative and therapeutic regimens for recurrence to be detailed subsequently, the previous concerns regarding the use of an LD graft may not be appropriate. However, the recurrence of NS/FSGS in two recipients with a homozygous podocin mutation who received an LD graft from their obligatory heterozygote mother indicates that the obligatory heterozygote parental donor may not be ideal [29, 30]. Similarly, the development of FSGS and ESRD in three donors [28, 31] at some point following donation also indicates that appropriate informed consent should be obtained prior to utilizing an LD graft in recipients with FSGS.

## What are the therapeutic options available to produce a remission following the recurrence of NS in recipients with FSGS?

In 1988, Laufer et al. [32] reported the remission of NS in two pediatric recipients following nine sessions of PP at 3 and 6 months post-transplant. Both recipients manifested prolonged ATN. This led to the general use of PP in patients with recurrence. However, no randomized control trial was ever performed and the rate of remission has been variable.

Ingulli et al. [33] in 1990 reported the success in inducing a remission with high-dose cyclosporine in three pediatric recipients. The rationale for the increased dosage was that hyperlipidemia bound the cyclosporine and higher doses were required to achieve adequate bioavailability. The precise dose and length of treatment with the “high-dose” regimen were never delineated in a controlled trial. However, this report led to the general approach of using a higher maintenance dosage of cyclosporine once a remission was obtained.

Recently, Raafat et al. [34] reported a recurrence rate of 67% (16/24) in recipients with FSGS between 1991 and 2003. The oral dose of cyclosporine was increased to 24 mg/kg/day until either remission was achieved or renal toxicity was evident. Complete remission was achieved in 69% (11/16) within 14 days to 19 months (10/11 within 3 months) and 12% (2/16) had a partial remission. Concomitant PP was administered to 7/16 recipients with recurrence.

Cochat et al. [35] in 1993 introduced a regimen of PP and cyclophosphamide in three patients who remitted within 12–24 days. This regimen has been adopted by some centers, especially for patients who failed to remit with PP alone. However, again, no controlled trial has been performed.

Plasma protein absorption was introduced by Dantal et al. [36] in 1994 for recipients with an identified circulating factor. Although proteineuria was reduced in the eight patients treated, it was permanent in only one patient following discontinuation of the procedure. Subsequent reports utilizing this technique in pediatric recipients are limited.

Recently, Salomon et al. [37] reported an 82% (14/17) remission rate utilizing IV Cyclosporine at 3 mg/kg/day to reach a cyclosporine level of 250–350 mg/dl. These data add to the potential utility of high-dose cyclosporine in inducing a remission following the recurrence of NS/FSGS.

With the use of angiotensin-converting enzyme/angiotensin receptor blocker (ACE/ARB) therapy to reduce proteinuria in patients with various forms of renal disease, it would seem logical to extend the use of these agents to recipients with the recurrence of NS/FSGS. However, there has been no controlled study of the use of these drugs either alone or as adjunctive to other therapeutic regimens.

## Can pre-operative PP reduce the risk of recurrence of NS/FSGS?

Ohta et al. [38] in 2001 reported the recurrence of NS/FSGS in 9/21 grafts. Pre-operative PP was performed in 15 and recurrence developed in five, whereas 4/6 without pre-operative PP developed recurrence. The authors concluded that pre-operative PP was effective in preventing the recurrence of NS/FSGS.

At the International Pediatric Transplant Association (IPTA) Congress in August 2005, Rainthavorn et al. [39] from the UCLA reported a recurrence rate of 100% in three recipients of DD grafts who received one session of pre-operative PP, whereas it was 57% (4/7) in recipients of LD grafts who received <5 pre-operative PP and 0% in three recipients who received >5 pre-transplant PP sessions. The authors concluded that >5 pre-operative PP sessions prevented the recurrence of NS/FSGS.

Kawamura et al. [40], at the same IPTA congress, reported a recurrence rate of 15.4% (2/13) in LD recipients who received 1–2 pre-operative PP sessions, as well as native kidney nephrectomy. Recurrence was evident at 6 days and 8 months post-transplant, respectively, and remitted with post-transplant PP.

A recent publication involving primarily adult recipients (9/10 were adult) utilized a combination of pre- and post-operative PP in high-risk patients—six with recurrence in a prior graft and four with rapid progression to ESRD (<3 years) [7]. The LD recipients received eight courses of PP over a two-week period—one week prior to and one week following transplantation. The DD recipients initiated PP within 24 hours of transplantation and continued for two weeks post-transplant. There was no recurrence in the four recipients with rapid progression to ESRD and 3/6 recipients with a recurrence in a prior graft had no recurrence.

Certainly, the utilization of pre-operative and prophylactic post-operative PP appears promising in high-risk patients. Controlled multicenter trials are warranted to delineate the optimal preventative and therapeutic approaches.

## Conclusions

The incidence of recurrence of nephrotic syndrome/focal segmental glomerulosclerosis (NS/FSGS) has remained at ~30% for the past four decades, despite the introduction of newer and more potent immunosuppressive regimens. Multiple risk factors have been identified to predispose or precipitate clinical recurrence. However, no prospective multicenter studies have validated the relationships. Mutations of the gene encoding for podocin may identify a population of patients with NS/FSGS who are at a lower risk for clinical recurrence. Because of the potential for an increased incidence of recurrence and/or graft loss, living donor (LD) grafts should be used with constraint. Graft survival rates in recipients with NS/FSGS are decreased. However, if a remission can be induced, the outlook for long-term graft survival is substantially improved.

Various therapeutic regimens, including pre-operative plasmapheresis (PP), high-dose cyclosporine, IV cyclosporine, and adjunctive cyclophosphamide, have proven to be effective. However, there have been no multicenter controlled studies to delineate the optimal therapeutic approach. Recent use of pre-operative and/or post-operative prophylactic PP to prevent the recurrence of NS/FSGS is exciting. Controlled trials are required to validate efficacy and delineate a precise preventative regimen.

### Questions

(Answers appear following the references)

1. The incidence of recurrence of FSGS is:
  - Comparable in whites and African-Americans
  - Less in whites than Hispanics
  - Less with re-transplants than with primary grafts
  - Not influenced by pre-transplant plasmapheresis
  - Between 30% and 40%
2. The use of a deceased donor is mandatory in a patient with FSGS when:
  - A living donor graft is not available
  - The initial graft was lost as a result of recurrence
  - The parental donor is not an obligatory podocin heterozygote
  - The potential live-donor is a haplotype mismatch
  - The potential live-donor is a B-antigen mismatch
3. Graft survival following the recurrence of NS in a patient with FSGS is dependent upon:
  - A reduction in the magnitude of proteinuria to 3.0 g/kg/day
  - The lack of histologic evidence of FSGS during the initial post-transplant month
  - Inducing a complete remission of the NS
  - The immediate use of post-transplant plasmapheresis
  - The use of a one haplotype-matched live-related donor
4. The optimal approach to prevent the recurrence of NS in patients with FSGS:
  - Includes cyclophosphamide and plasmapheresis
  - Includes one week of pre-transplant plasmapheresis
  - Includes post-operative tacrolimus and plasmapheresis
  - Includes post-operative sirolimus and plasmapheresis
  - Has not been delineated
5. The recurrence of NS in patients with congenital nephrotic syndrome:
  - Has not been reported with the “Finnish” type
  - Is >80% in recipients with diffuse mesangial sclerosis
  - Is uniform with Fin-major/Fin-minor genotype
  - Is associated with the development of antinephrin antibodies
  - Is associated with anti-podocin antibodies
6. Which of the following does not impact on the recurrence of NS in patients with FSGS:
  - Race
  - Native kidney nephrectomy
  - Recurrence in a prior graft
  - Podocin mutation
  - None of the above

## References

[1] NAPRTCS 2004 Annual Report. Available online at https://doi.org/web.emmes.com/study/ped/annlrept/annlrept2004.pdf

[2] Hoyer JR, Vernier RL, Najarian JS, Raij L, Simmons RL, Michael AF (1972). Recurrence of idiopathic nephrotic syndrome after renal transplantation. 1972. Lancet.

[3] (1972) Focal glomerulosclerosis. Lancet 2:367 4114727

[4] Tejani A, Stablein DH (1992). Recurrence of focal segmental glomerulosclerosis posttransplantation: a special report of the North American Pediatric Renal Transplant Cooperative Study. J Am Soc Nephrol.

[5] Baum MA, Stablein DM, Panzarino VM, Tejani A, Harmon WE, Alexander SR (2001). Loss of living donor renal allograft survival advantage in children with focal segmental glomerulosclerosis. Kidney Int.

[6] Jungraithmayr T, Clara A, Zimmerhackl L (2005). Recurrence of focal segmental glomerulosclerosis (FSGS) after renal transplantation: results of the European Collaborative FSGS transplantation study group (ECOFTS). Pediatr Transplant.

[7] Gohh RY, Yango AF, Morrissey PE, Monaco AP, Gautam A, Sharma M, McCarthy ET, Savin VJ (2005). Preemptive plasmapheresis and recurrence of FSGS in high-risk renal transplant recipients. Am J Transplant.

[8] Kerjaschki D (2001). Caught flat-footed: podocyte damage and the molecular bases of focal glomerulosclerosis. J Clin Invest.

[9] Bertelli R, Ginevri F, Caridi G, Dagnino M, Sandrini S, Di Duca M, Emma F, Sanna-Cherchi S, Scolari F, Neri TM, Murer L, Massella L, Basile G, Rizzoni G, Perfumo F, Ghiggeri GM (2003). Recurrence of focal segmental glomerulosclerosis after renal transplantation in patients with mutations of podocin. Am J Kidney Dis.

[10] Caridi G, Perfumo F, Ghiggeri GM (2005). NPHS2 (Podocin) mutations in nephrotic syndrome. Clinical spectrum and fine mechanisms. Pediatr Res.

[11] Howie AJ, Pankhurst T, Sarioglu S, Turhan N, Adu D (2005). Evolution of nephrotic-associated focal segmental glomerulosclerosis and relation to the glomerular tip lesion. Kidney Int.

[12] Ingulli E, Tejani A (1991). Racial differences in the incidence and renal outcome of idiopathic focal segmental glomerulosclerosis in children. Pediatr Nephrol.

[13] Baqi N, Tejani A (1997). Recurrence of the original disease in pediatric renal transplantation. J Nephrol.

[14] Bartosh SM, Stablein DM, Fine RN (2004) Recurrence of focal segmental glomerulosclerosis (FSGS) following pediatric kidney transplantation in the modern immunosuppression era: a report from the North American Pediatric Renal Transplant Cooperative Study (NAPRTCS). Submitted for presentation at the American Transplant Congress, Boston, Massachusetts, 15–19 May 2004

[15] Savin VJ, Sharma R, Sharma M, McCarthy ET, Swan SK, Ellis E, Lovell H, Warady B, Gunwar S, Chonko AM, Artero M, Vincenti F (1996). Circulating factor associated with increased glomerular permeability to albumin in recurrent focal segmental glomerulosclerosis. N Engl J Med.

[16] Raafat R, Travis LB, Kalia A, Diven S (2000). Role of transplant induction therapy on recurrence rate of focal segmental glomerulosclerosis. Pediatr Nephrol.

[17] Gagnadoux MF (2002). Ask the expert. Does antibody induction therapy with daclizumab or basiliximab increase the risk of recurrence of post-transplant focal segmental glomerulosclerosis?. Pediatr Nephrol.

[18] Sheth RD, Kale AS, Goldstein SL, Brewer ED (2001). Rapid recurrence of post-transplant FSGS in pediatric patients after daclizumab induction. Pediatr Nephrol.

[19] Hubsch H, Montane B, Abitbol C, Chandar J, Shariatmadar S, Ciancio G, Burke G, Miller J, Strauss J, Zilleruelo G (2005). Recurrent focal glomerulosclerosis in pediatric renal allografts: the Miami experience. Pediatr Nephrol.

[20] Odorico JS, Knechtle SJ, Rayhill SC, Pirsch JD, D’Alessandro AM, Belzer FO, Sollinger HW (1996). The influence of native nephrectomy on the incidence of recurrent disease following renal transplantation for primary glomerulonephritis. Transplantation.

[21] Baum MA, Ho M, Stablein D, Alexander SR, North American Pediatric Renal Transplant Cooperative Study (2002). Outcome of renal transplantation in adolescents with focal segmental glomerulosclerosis. Pediatr Transplant.

[22] Huang K, Ferris ME, Andreoni KA, Gipson DS (2004). The differential effect of race among pediatric kidney transplant recipients with focal segmental glomerulosclerosis. Am J Kidney Dis.

[23] Schachter AD, Harmon WE (2001). Single-center analysis of early recurrence of nephrotic syndrome following renal transplantation in children. Pediatr Transplant.

[24] Sweet SC, Wong HH, Webber SA, Horslen S, Guidinger MK, Fine RN, Magee JC (2006). Pediatric transplantation in the United States, 1995–2004. Am J Transplant.

[25] Cameron JS, Senguttuvan P, Hartley B, Rigden SP, Chantler C, Koffman G, Williams DG, Ogg CS (1989). Focal segmental glomerulosclerosis in fifty-nine renal allografts from a single centre; analysis of risk factors for recurrence. Transplant Proc.

[26] Senggutuvan P, Cameron JS, Hartley RB, Rigden S, Chantler C, Haycock G, Williams DG, Ogg C, Koffman G (1990). Recurrence of focal segmental glomerulosclerosis in transplanted kidneys: analysis of incidence and risk factors in 59 allografts. Pediatr Nephrol.

[27] First MR (1995). Living-related donor transplants should be performed with caution in patients with focal segmental glomerulosclerosis. Pediatr Nephrol.

[28] Winn MP, Alkhunaizi AM, Bennett WM, Garber RL, Howell DN, Butterly DW, Conlon PJ (1999). Focal segmental glomerulosclerosis: a need for caution in live-related renal transplantation. Am J Kidney Dis.

[29] Ruf RG, Lichtenberger A, Karle SM, Haas JP, Anacleto FE, Schultheiss M, Zalewski I, Imm A, Ruf EM, Mucha B, Bagga A, Neuhaus T, Fuchshuber A, Bakkaloglu A, Hildebrandt F, Arbeitsgemeinschaft Fur Padiatrische Nephrologie Study Group (2004). Patients with mutations in NPHS2 (podocin) do not respond to standard steroid treatment of nephrotic syndrome. J Am Soc Nephrol.

[30] Weber S, Gribouval O, Esquivel EL, Moriniere V, Tete MJ, Legendre C, Niaudet P, Antignac C (2004). NPHS2 mutation analysis shows genetic heterogeneity of steroid-resistant nephrotic syndrome and low post-transplant recurrence. Kidney Int.

[31] Ismail-Allouch M, Burke G, Nery J, Roth D, Esquenazi V, Ruiz P, Miller J (1993). Rapidly progressive focal segmental glomerulosclerosis occurring in a living related kidney transplant donor: case report and review of 21 cases of kidney transplants for primary FSGS. Transplant Proc.

[32] Laufer J, Ettenger RB, Ho WG, Cohen AH, Marik JL, Fine RN (1988). Plasma exchange for recurrent nephrotic syndrome following renal transplantation. Transplantation.

[33] Ingulli E, Tejani A, Butt KM, Rajpoot D, Gonzalez R, Pomrantz A, Ettenger R (1990). High-dose cyclosporine therapy in recurrent nephrotic syndrome following renal transplantation. Transplantation.

[34] Raafat RH, Kalia A, Travis LB, Diven SC (2004). High-dose oral cyclosporine therapy for recurrent focal segmental glomerulosclerosis in children. Am J Kidney Dis.

[35] Cochat P, Kassir A, Colon S, Glastre C, Tourniaire B, Parchoux B, Martin X, David L (1993). Recurrent nephrotic syndrome after transplantation: early treatment with plasmaphaeresis and cyclophosphamide. Pediatr Nephrol.

[36] Dantal J, Bigot E, Bogers W, Testa A, Kriaa F, Jacques Y, Hurault de Ligny B, Niaudet P, Charpentier B, Soulillou JP (1994). Effect of plasma protein adsorption on protein excretion in kidney-transplant recipients with recurrent nephrotic syndrome. N Engl J Med.

[37] Salomon R, Gagnadoux MF, Niaudet P (2003). Intravenous cyclosporine therapy in recurrent nephrotic syndrome after renal transplantation in children. Transplantation.

[38] Ohta T, Kawaguchi H, Hattori M, Komatsu Y, Akioka Y, Nagata M, Shiraga H, Ito K, Takahashi K, Ishikawa N, Tanabe K, Yamaguchi Y, Ota K (2001). Effect of pre-and postoperative plasmapheresis on posttransplant recurrence of focal segmental glomerulosclerosis in children. Transplantation.

[39] Rianthavorn P, Bhakta N, Gjertson D, Ettenger R (2005). A coherent approach to recurrent focal segmental glomerulosclerosis in children? The effects of high dose cyclosporine and pretransplant plasmapheresis. Pediatr Transplant.

[40] Kawamura T, Motoyama O, Aikawa A, Ohara T, Motoyama O, Hasegawa A (2005). Outcome of first living related renal transplantation for pediatric patients with focal segmental glomerulonephritis in Japan—a single center experience. Pediatr Transplant.

